# Understanding child development and care integrality: Primary Health Care doctors and nurses’ view

**DOI:** 10.1590/1984-0462/2024/42/2023127

**Published:** 2024-04-29

**Authors:** Catarina Falleiros Nogueira Rojas, Danielle Abdel Massih Pio, Ana Carolina Nonato

**Affiliations:** a Faculdade de Medicina de Marilia, Marília, SP, Brasil.

**Keywords:** Child development, Primary health care, Comprehensive health care, Desenvolvimento infantil, Atenção primária à saúde, Assistência integral à saúde da criança

## Abstract

**Objective:**

To identify perceptions of primary care health professionals regarding the conceptual aspects of child development and propose strategies to address difficulties.

**Methods:**

This descriptive-analytical study was conducted in a small municipality in the countryside of the State of São Paulo, Brazil. The primary health care in this region is comprised of Family Health Units and Basic Health Units. The sample included 52 participants, consisting of doctors and primary care nurses. A questionnaire with open and closed questions was utilized, covering knowledge and practices related to child development. For this study, the first question of the questionnaire, which asked for a descriptive response about participants’ understanding of child development, was employed. The responses were transcribed, and content analysis using the thematic approach was conducted.

**Results:**

Among the participants, 54% were nurses, and the average duration of working with the pediatric population was ten years. 80% reported never having undergone training in child development. The analysis of the responses revealed heterogeneity in the professionals’ understanding of the conceptual dimension of child development. Additionally, there was an insufficient grasp of the theoretical and practical aspects and a scarcity of resources to support comprehensive care for children. A predominant biomedical model focusing on disease and biological aspects of child health was evident in defining the understanding of the subject.

**Conclusions:**

The findings underscore the necessity of implementing health education initiatives and service projects in primary care settings. It is crucial to strengthen a comprehensive perspective of child health within the biopsychosocial model of the health-disease process.

## RESUMO

**Objetivo:**

Identificar percepções de profissionais de saúde da atenção primária acerca dos aspectos conceituais do desenvolvimento infantil e propor estratégias de enfrentamento das dificuldades.

**Métodos:**

Estudo descritivo-analítico conduzido em um município de pequeno porte no interior do estado de São Paulo, Brasil, cuja atenção primária é composta das Unidades de Saúde da Família e Unidades Básicas de Saúde. A amostra foi constituída por 52 participantes, entre médicos e enfermeiros da atenção primária. Utilizou-se um questionário com perguntas abertas e fechadas, abordando conhecimentos e práticas sobre desenvolvimento infantil. Para este estudo, foi utilizada a primeira pergunta do questionário, de natureza dissertativa, que abordou como os participantes compreendiam o desenvolvimento infantil. As respostas foram transcritas e foi utilizada a técnica de análise de conteúdo na modalidade temática.

**Resultados:**

Dos participantes, 54% eram enfermeiros, e a média de tempo de trabalho com a faixa pediátrica foi de dez anos. Referiram nunca ter realizado curso de formação em desenvolvimento infantil 80%. Quanto à análise das respostas, observou-se heterogeneidade no que se refere à dimensão conceitual do tema por parte dos profissionais, além de uma apreensão teórico-prática insuficiente e escassez de recursos que fortaleçam um atendimento integral à criança. Prevalece um modelo biomédico centrado na doença e aspectos biológicos da saúde infantil quando definem a compreensão sobre o tema.

**Conclusões:**

Os dados apontam para a necessidade de implementação de projetos de educação em saúde e em serviço na atenção primária. Faz-se premente o fortalecimento de um olhar integral à saúde da criança sob o modelo biopsicossocial do processo saúde-doença.

## INTRODUCTION

Child development encompasses a complex and multifaceted system of continuous growth and acquisition of human skills. It involves intricate hierarchical and dynamic mechanisms that facilitate adaptive socialization and transform individuals into active participants within their socio-historical environment.^1,2^

Child development is influenced by multiple aspects, including physical-motor abilities, cognitive functions (such as reasoning and memory), affective experiences (how individuals perceive and respond emotionally), and social interactions (relationships with others and cultural factors).^3^

The study of child development has been influenced by theoretical models from both the natural and biological sciences, as well as the human and social sciences. One significant theoretical framework that allows for a comprehensive understanding of development within a biopsychosocial model of child health is the Bioecological Theory of Human Development.^4^ This theory provides a foundation for examining the conceptual aspects of child development in a comprehensive manner.

According to this, development consists of four interrelated elements known as the process-person-context-time (PPCT) model. In this model, the “process” refers to the interactions and relationships between the individual and their surrounding context. The “person” represents the innate characteristics and attributes of the individual. The “context” is broadly understood and encompasses various levels, including the microsystem, exosystem, mesosystem, and macrosystem.^4,5^

The microsystem refers to the immediate environment of the child, such as the family. The mesosystem includes the interrelationships between two or more microsystems that the child is a part of, such as the connections between the family and school microsystems, or the relationship between the family and the child’s friends. The exosystem comprises external influences that indirectly impact the child, such as the parents’ work environment. The macrosystem relates to the broader cultural and societal factors that influence the community, including religion, politics, economics, and public health policies.^4^

**Figure 1:**
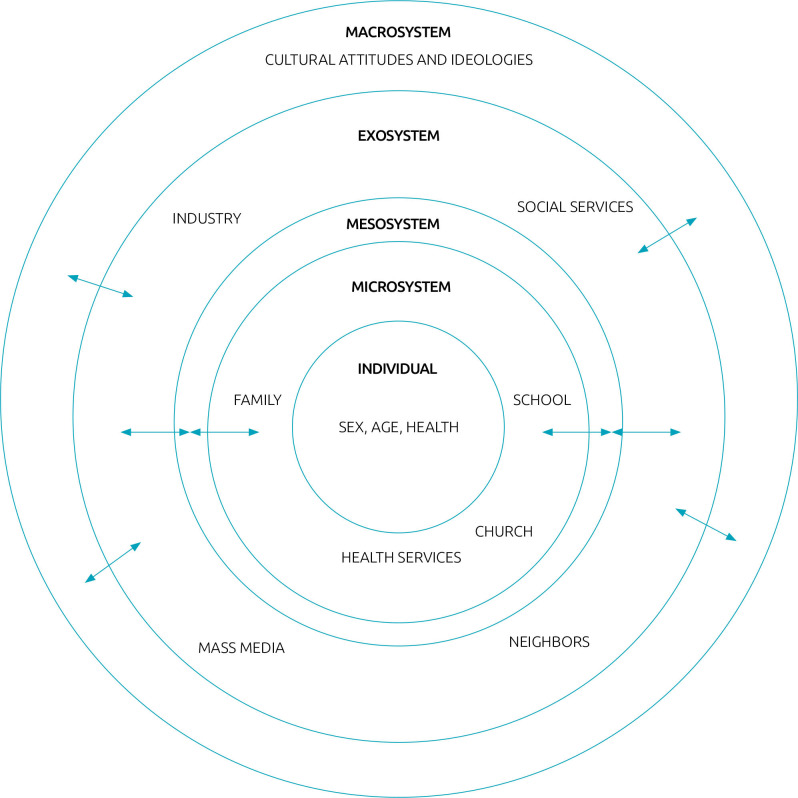
Person-Process-Context-Time Model (Bioecological Theory of Human Development).^6^

Legend: 1) Microsystem: The innermost level containing the developing person’s immediate environments and interpersonal relationships (e.g., family, school, neighborhood, peers). This is considered the gravitational center where proximal processes occur. 2) Mesosystem: The connections and interactions between two or more microsystem environments (e.g., connections between family and school). 3) Exosystem: External environments that indirectly influence the developing person (e.g., parent’s workplace, community resources).4) Macrosystem: The overarching culture, values, laws, and customs that influence all other subsystems and the developing person. Arrows connecting the systems represent the bi-directional influences between the developing individual and the interconnected systems over the course of development. Central to this framework is the interaction between characteristics of the developing person and the various levels of environmental context.

Additionally, in the PPCT model,^4^ “time” serves as a moderator of changes throughout an individual’s lifespan. These interconnected elements and their relationship are illustrated in Figure 1.^4,6^

The healthcare professionals responsible for providing childcare services in primary healthcare units should be able to identify the most vulnerable and at-risk children. They should also offer appropriate anticipatory guidance for the various stages of development, direct parents to additional sources of guidance and practical resources and collaborate in implementing safety-promoting measures.^7^

In terms of the responsibilities of healthcare professionals, namely doctors and nurses, in the systematic provision of pediatric care in primary healthcare settings, it is crucial to emphasize the significance of having a solid theoretical and practical understanding of child development in order to properly evaluate cases and prevent delays in interventions.^8^

The identified problem, supported by scientific publications, lies in the constant undervaluation of these practices as fundamental aspects of pediatric care in primary healthcare.^9-12^ This issue partly arises from challenges in providing care and a lack of preparedness in problem identification among medical professionals. Additionally, the scarcity of resources for training further exacerbates the problem, leading to a hierarchical organization with a heavy reliance on referrals, references, and counter-referrals, as well as bureaucratic processes with limited effectiveness.

Consequently, what becomes evident is the delayed referral of cases to specialized outpatient clinics, resulting in delayed diagnoses and missed opportunities for effective interventions. However, it is important to note that the vulnerability of early childhood is balanced by the remarkable neuronal plasticity during this period, underscoring the urgent need for early detection to enhance prognostic possibilities.

Considering the healthcare professionals involved in providing childcare services in municipal settings, the objective of this study was to identify perceptions of primary care physicians and nurses regarding child development. The bioecological model, specifically the process-person-context-time concept, was utilized as a reference for this analysis.

## METHOD

This descriptive-analytical study was conducted in the primary care setting of Marília city, located in the interior of the State of São Paulo, which currently has 12 pediatricians and 28 nurses distributed across 9 traditional Basic Health Units (UBS), and approximately 45 doctors and 52 nurses in 43 Family Health Strategy Units (ESF). The inclusion criteria were being a healthcare professional who admits and provides follow-up care for children in pediatric consultations (physicians and nurses within the scope of the Unified Health System) and who work with pediatric care in primary healthcare in the municipality. Professionals who have exclusive employment ties with private healthcare institutions or who were on vacation or on leave during the questionnaire administration period were excluded.

The estimated population was 126 doctors and nurses, and an exhaustive sampling of 52 participants was obtained despite the questionnaire being available with an open link online throughout the data collection period, and the repeated phone contacts weekly with the teams and nurses responsible for the units, with encouragement from the municipal department. The clinical setting was composed of five UBS and 25 USFs.

Data collection was performed using a questionnaire consisting of 14 questions, including both open-ended and multiple-choice questions. The questionnaire covered various aspects such as conceptual and theoretical-practical aspects of child development, evaluation tools utilized in child development surveillance, aspects related to the care network, and suggestions for professional improvement in the field. To maintain participants’ identities secure, each transcript below was identified by a code: ‘P’ and a number corresponding to each participant.

For this study, we will focus on analyzing the data from the first question of the questionnaire, which required participants to provide a discursive response regarding their understanding of child development. The analysis of this question was conducted using the Content Analysis technique in the thematic modality.^13^

## RESULTS

The sociodemographic characteristics of the participants indicated that over 90% of them worked in the public sector, with 7.7% working in both the public and private sectors. Among the participants, 54% were nurses. On average, the participants had thirteen years of training. The majority, more than 90%, worked in the public sector, with a weekly workload of approximately twenty-one hours. When asked about their experience in childcare, the average was 10.2 years. In terms of the weekly workload in the pediatric area, the average reported was 21.4 hours. Furthermore, 80.8% of the participants stated that they had never received vocational training in child development. Only eight nurses and one doctor had training in child development, without any other specializations in the field, and two doctors were pediatricians.

As for the answers to the questionnaire, it was found that 6% of the participants did not routinely ask the parents about child development, 25% of the professionals did not measure the head circumference in all pediatric consultations, and 92% only asked about the child’s language. As for the validated instruments for assessing child development, 48% did not complete the Child Handbook of the Ministry of Health. Of the 52 respondents, only 3 knew and applied the Denver II scale, and less than 10% reported knowing and applying the M-CHAT scale for autism screening. More than 80% of the participants responded that, on rare occasions, complaints of delays are resolved with Primary Health Care resources, and the main difficulties pointed out are lack of structure, materials and complete teams.

The qualitative analysis of the results was conducted in two stages. Firstly, a thorough reading of the transcribed interviews was performed, selecting ideas that stood out in the responses and were related to the study’s objective, organizing them into core themes. The second stage involved the development of themes based on the research objectives, through the classification and organization of the gathered information.

The data analysis of the research was categorized into four themes:

1. Different understandings of child development;
2. Assessment of child development: use of tools and guidance for caregivers;
3. Challenges Regarding Child Development Care, and
4. Training spaces for child development surveillance: potentials and challenges.

Now, we will focus on the first theme, “Different understandings of child development”, which was built upon the formation of two core meanings: “The understanding of child development as a process of progressive acquisition of skills” and “The understanding of child development as an interface of growth”.

It was observed that some respondents understood the concept of child development in a multidimensional manner, viewing it as a continuous, interdisciplinary, and hierarchical process.

*“Phase in which the child acquires and enhances cognitive, motor, social and emotional skills”. (P1)*

On the other hand, participants demonstrated varied understandings of child development. Some primarily associated it with motor development, while others emphasized the development of cognitive abilities or socio-emotional aspects.

*“Development of cognitive functions is proceeding as expected”. (P51)*

Throughout the answers, it is evident that professionals use terms that reflect their understanding of the theme, such as “changes”, “learning process”, “phases”, “milestone acquisition”, and “maturation”. However, it is also noticeable that participants themselves used the same term to conceptualize child development, which may suggest difficulties in fully comprehending the definition.

*“Psychomotor development of the child”. (P47)*

The understanding of development was often associated with physiological processes and somatic aspects. Parameters such as weight-height gain, hygiene, and nutrition were mentioned as indicators. Additionally, terms such as physical growth and biological changes were used.

For some professionals, the biomedical model, which focuses on disease and biological aspects of child health, still appears to prevail when defining their understanding of the subject. This is reflected in their emphasis on weight-stature gain and vaccination in their care practices. It is unclear from some of the responses whether these characteristics align with the understanding of patients’ needs within the biopsychosocial paradigm.

*“Weight gain, reflexes, muscle strength, vision, hearing, hygiene…” (P18)*

In the overall analysis of the responses, it can be observed that, while professionals grasp the concept as a dynamic and progressive movement, it is not clear whether they understand human development within a bioecological model. This model, integrated with the components of person-process-context-time, would result in a greater availability of resources to modify an environment where individuals can autonomously take advantage of their opportunities and have a sense of belonging to the value systems of their social context.

## DISCUSSION

The data analysis revealed a clear gap in understanding child development, with variations in their comprehension leaning heavily towards either physiological processes or cognitive abilities. These findings reflect a disconnection from the broader bioecological model of child development, which emphasizes the interactions and relationships between the child and their environment, considering personal, contextual, and temporal elements.^4,5^

Parallels can be found in literature. For instance, three studies collectively indicate a variation in the comprehension and practices of child development among healthcare professionals. A conceptual analysis revealed that trained professionals tended to give more weight to environmental factors than to individual aspects of development delays, indicating an understanding of the complexity of child development.^5^ Similarly, in primary care settings, child development was often seen through a biological lens, with an emphasis on anthropometric measurements.^14^ These findings are in contrast with a qualitative assessment finding that neuropsychomotor evaluations are generally considered part of care, but a thorough assessment that includes neurological milestones and risk signs was often absent.^15^ However, an intervention study indicated that training workshops could improve knowledge and practices around developmental surveillance.^16^ The differences seem to hinge on the depth of understanding and approach towards child development, and the shared point is the need for more comprehensive understanding and training in child development for healthcare professionals.

It is well-established that the period of early childhood, from birth to six years of age, is crucial for the foundation of human development and adaptive behavior. This phase is considered the most formative in a person’s life,^1,17^ as it encompasses various maturation processes and the formation of new neuronal circuits that contribute to adaptive socialization.^2,18,19^

Advancements in neuroscience and longitudinal studies have led to the understanding that adverse experiences during childhood have long-term negative effects on the developing brain.^20,21^ Intervention studies involving children exposed to adverse conditions have demonstrated significant results in areas such as intelligence quotient, educational attainment, income distribution, health biomarkers, reduction of violence, and psychological disorders, including multigenerational effects.^22^

Recent global data indicates a rise in the prevalence of neurodevelopmental disorders, specifically Autism Spectrum Disorder, which currently affects one in 36 eight-year-old children. These findings prompt reflection on how healthcare professionals understand developmental delays and, consequently, the concept of child development itself.^23^

Despite being a universally recognized topic, with a growing number of publications recently that have presented new scientific evidence for interventions and program implementation,^24^ the evaluation of child development continues to be undervalued in childcare consultations and by the government. There is a lack of proactive action from professionals who serve as the first point of contact for at-risk children.^14,16,24-28^

Despite being regulated in the 1990s, child development surveillance has not yet become a fully integrated practice in primary healthcare. Data indicates a lack of significant progress globally in addressing developmental disabilities, even though infant mortality rates have decreased by half between 1990 and 2016.^29^ This can be attributed to the absence or inadequacy of systematic policies and interventions to meet the needs of individuals who survive childhood illnesses but lack the neuropsychomotor skills required for independent adulthood.^25-27,30^

The previously reported fragmented and insufficient understanding, amplified by the heterogeneity of the evaluation in childcare and the use of not systematized tools, could lead to a lack of accuracy in detecting warning signs, determinants, or health needs in children addressed in these services. The results point to failures in identifying risk criteria by professionals who claim to never or rarely treat patients with such complaints, considering that the understanding of normal development itself in its conceptual dimension is not yet well established. This promotes inaccuracies and underestimation of these elements as part of the pediatric consultation in Primary Health Care.

As such, strengthening the competencies of professionals and applying this knowledge in their interactions with users can contribute to the creation of systems that foster a more dialogical relationship. This entails recognizing the macro-level determinants that directly influence the relationships established between families and children, with the goal of providing responsive and appropriate care.^31^

This study aimed to identify how primary care professionals perceive the concept of child development and whether there is a need to implement projects that bring visibility to this topic. The results obtained from the responses indicate that the understanding of child development lacks transformative meaning and a comprehensive knowledge base that incorporates purpose. There is a need for more integrated and critically reflective conceptions of the processes and outcomes associated with child development, which would enable children to modify their environments and optimize their opportunities with autonomy and a sense of belonging to their social context’s value systems.

There is the perception of formulating vague and general answers that prompt the recognition of the need to build a more comprehensive knowledge and that incorporates a purpose, as a way of subsidizing still incomplete conceptions, little integrated to a critical reflection on the processes and their outcomes regarding the proposed theme.

Based on the bioecological concept, this study reveals a heterogeneity in the conceptual understanding of child development among doctors and nurses engaged in childcare. The definitions vary, ranging from a reductionist and fragmented model to a more comprehensive and holistic perspective. Most professionals lack a focus on the social determinants that extend beyond the preventive and curative aspects of development. There is a need for critical reflection on the processes and outcomes associated with child development within the proposed theme.

Furthermore, the understanding of child development is not connected to a transformative process of meanings or a greater availability of resources that would empower children to modify their environment and embrace opportunities autonomously, while recognizing their belonging to the social context’s value systems.

Overall, this study aims to shed more light concerning child development. It seeks to contribute to the search for solutions that facilitate actions promoting normal development within an intersectoral paradigm. This includes early identification of at-risk children and timely therapeutic interventions for potential conditions. The implementation of social pediatrics as a new field of knowledge and practice is emphasized, supported by a systemic model of child health, and incorporating proposed reforms in medical and nursing education.

In addition, initiatives focused on ongoing and continuous training for health professionals working with early childhood can foster the creation of collective spaces for discussion, strengthen teamwork, and facilitate the integration of teaching and service through collaborative knowledge-building in the work environment. This ensures comprehensive and holistic care.

The study, while instrumental in providing insight into the surveillance of child development in the municipal context, faced several limitations that warrant discussion to contextualize the findings accurately. Firstly, the sample size is a significant constraint, even after a continuous effort from researchers to increase it. The selection of only 52 participants from the total pool of professionals in the city may not adequately represent the actual practices, knowledge, and attitudes prevalent among the wider professional community. This small sample limits the capability to generalize the findings to a broader context. However, it can be used as a starting point to other studies with a broader sample size. Furthermore, there is an inherent limitation in the selection of participants who filled out the questionnaire. By relying on self-selection, the study may have engaged professionals who do not have specialized qualifications or training on child development surveillance. This opens the possibility that the perspectives of those who are potentially more informed yet did not participate in the survey are missing from the study. Another possible limitation is that focusing only on one theme from the four categories recognized in the quantitative analysis further narrows the research extent. This limited scope narrows the research narrative and potentially overlooks the multifaceted aspects of child development surveillance that could have been captured through a broader thematic investigation. On the other hand, it was necessary to provide a detailed analysis on this, considering the main core of this study.

## Data Availability

The database that originated the article is available with the corresponding author.

## References

[1] Miguel PM, Pereira LO, Silveira PP, Meaney MJ (2019). Early environmental influences on the development of children’s brain structure and function. Dev Med Child Neurol.

[2] Brown KA, Parikh S, Patel DR (2020). Understanding basic concepts of developmental diagnosis in children. Transl Pediatr.

[3] Faria AR (1998). Desenvolvimento da criança e do adolescente segundo Piaget.

[4] Tudge J, Moreira L, Carvalho AM (2008). Família e educação: olhares da Psicologia.

[5] Souza JM, Veríssimo MLOR (2015). Child development: analysis of a new concept. Rev Lat Am Enfermagem.

[6] Veiga GR, Silva GA, Padilha BM, Lima MC (2023). Determining factors of child linear growth from the viewpoint of Bronfenbrenner’s Bioecological Theory. J Pediatr (Rio J).

[7] Brazil. Ministério da Saúde (2018). Política nacional de atenção integral à saúde da criança: orientações para implementação.

[8] Brazil. Ministério da Saúde (2016). Diretrizes de estimulação precoce crianças de zero a 3 anos com atraso no desenvolvimento neuropsicomotor.

[9] Leal SL, Oliveira ER, Pessoa ML (2022). Uso da caderneta de saúde da criança no acompanhamento do crescimento – uma revisão de escopo. Rev APS.

[10] Araújo BC, Gerzson LR, Almeida CS (2020). Aspectos avaliativos do desenvolvimento infantil na atenção básica: uma revisão integrativa. Arch Health Sci.

[11] Zeppone SC, Volpon LC, Del Ciampo LA (2012). Monitoramento do desenvolvimento infantil realizado no Brasil. Rev Paul Pediatr.

[12] Gaiva MA, Monteschio CA, Moreira MD, Salge AK (2018). Avaliação do crescimento e desenvolvimento infantil na consulta de enfermagem. Av Enferm.

[13] Bardin L (2016). Análise de conteúdo.

[14] Marinus MW, Silva LA, Santana JC, Soares AK, Barros MS (2022). Knowledge and practices of professionals of the family health strategy on promoting child development.

[15] Vieira DS, Dias TK, Pedrosa RK, Vaz EM, Collet N, Reichert AP (2019). Work process of nurses in child development surveillance. Reme Rev Min Enferm.

[16] Reichert AP, Collet N, Eickmann SH, Lima MC (2015). Child development surveillance: intervention study with nurses of the Family Health Strategy. Rev Latino-Am Enfermagem.

[17] Black MM, Walker SP, Fernald LC, Andersen CT, DiGirolamo AM, Lu C (2017). Early childhood development coming of age: science through the life course. Lancet.

[18] Walker SP, Wachs TD, Grantham-McGregor S, Black MM, Nelson CA, Huffman SL (2011). Inequality in early childhood: risk and protective factors for early child development. Lancet.

[19] Walker SP, Wachs TD, Gardner JM, Lozoff B, Wasserman GA, Pollitt E (2007). Child development: risk factors for adverse outcomes in developing countries. Lancet.

[20] Quezada S, Castillo-Melendez M, Walker DW, Tolcos M (2018). Development of the cerebral cortex and the effect of the intrauterine environment. J Physiol.

[21] Norbom LB, Ferschmann L, Parker N, Agartz I, Andreassen OA, Paus T (2021). New insights into the dynamic development of the cerebral cortex in childhood and adolescence: integrating macro- and microstructural MRI findings. Prog Neurobiol.

[22] Perkins JM, Kim R, Krishna A, McGovern M, Aguayo VM, Subramanian SV (2017). Understanding the association between stunting and child development in low- and middle-income countries: next steps for research and intervention. Soc Sci Med.

[23] Maenner MJ, Shaw KA, Bakian AV, Bilder DA, Durkin MS, Esler A (2021). Prevalence and characteristics of autism spectrum disorder among children aged 8 years — autism and developmental disabilities monitoring network, 11 sites, United States, 2018. MMWR Surveill Summ.

[24] A systematic review of parenting programmes for young children in low and middle income countries (2014).

[25] Engle PL, Fernald LC, Alderman H, Behrman J, O’Gara C, Yousafzai A (2011). Strategies for reducing inequalities and improving developmental outcomes for young children in low-income and middle-income countries. Lancet.

[26] Richter LM, Daelmans B, Lombardi J, Heymann J, Boo FL, Behrman JR (2016). Investing in the foundation of sustainable development: pathways to scale up for early childhood development. Lancet.

[27] Daelmans B, Darmstadt GL, Lombardi J, Black MM, Britto PR, Lye S (2017). Early childhood development: the foundation of sustainable development. Lancet.

[28] Roia A, Paviotti E, Ferluga V, Montico M, Monasta L, Ronfani L (2014). Promoting effective child development practices in the first year of life: does timing make a difference?. BMC Pediatr.

[29] Global Research on Developmental Disabilities Collaborators (2018). Lancet Glob Health.

[30] Yánez L, Jones CV (2018). Latin America and the potential of nurturing care framework as a platform for scale.

[31] Tan NQ, Cho H (2019). Cultural appropriateness in health communication: a review and a revised framework. J Health Commun.

